# Foundations of Ecological and Evolutionary Change

**DOI:** 10.1002/ece3.72454

**Published:** 2025-11-12

**Authors:** A. Bradley Duthie, Victor J. Luque

**Affiliations:** ^1^ Department of Biological and Environmental Sciences University of Stirling Stirling UK; ^2^ Department of Philosophy University of Valencia Valencia Spain

**Keywords:** eco‐evolutionary theory, ecology, evolution, fundamental theorem, population growth, price equation

## Abstract

Biological evolution is realised through the same mechanisms of birth and death that underlie change in population density. The deep interdependence between ecology and evolution is well established, and recent models focus on integrating eco‐evolutionary dynamics to demonstrate how ecological and evolutionary processes interact and feed back upon each other. Nevertheless, a gap remains between the logical foundations of ecology and evolution. Population ecology and evolution have fundamental equations that define how the size of a population (ecology) and the average characteristic within a population (evolution) change over time. These fundamental equations are a complete and exact description of change for any closed population, but how they are formally linked remains unclear. We link the fundamental equations of population ecology and evolution with an equation that sums how individual characteristics interact with individual fitness in a population. From this equation, we derive the fundamental equations of population ecology and evolutionary biology (the Price equation). We thereby identify an overlooked bridge between ecology and biological evolution. Our unification formally recovers the equivalence between mean population growth rate and evolutionary fitness and links this change to ecosystem function. We outline how our framework can be used to further develop eco‐evolutionary theory.

## Graphical abstract

We link the fundamental equations of population ecology and evolution with an equation that sums how individual characteristics interact with individual fitness in a population. From this equation, we derive the fundamental equations of population ecology and evolutionary biology (the Price equation). Our unification formally recovers the equivalence between mean population growth rate and evolutionary fitness and links this change to ecosystem function.

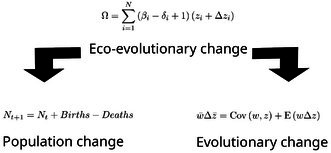

## 1 Introduction

Theoretical unification is a powerful tool for scientific advancement. Such unification has been a major goal in scientific research throughout history (Smocovitis 1992; Kitcher 1993), and its value is perhaps most evident in reconciling unconnected models and revealing new and unexpected empirical predictions. In evolutionary biology, the Price equation (Box 1) provides a unifying framework for evolutionary theory by exhaustively and exactly describing evolutionary change for any closed population (Price 1970; Luque 2017; Lehtonen et al. 2020). The Price equation is therefore fundamental, in the sense that it binds together all of evolutionary theory by formally defining what evolutionary change is and is not (Price 1970; Rice 2004; Gardner 2008; Frank 2017; Luque 2017; Luque and Baravalle 2021). Using this formal definition, the scope of, and relationships among, sub‐disciplines within evolutionary theory can be clarified. For example, fundamental equations of both population and quantitative genetics can be derived from the Price equation (Queller 2017). This provides conceptual clarity by demonstrating the logical consistency of different theoretical frameworks within evolutionary biology. Our aim here is to propose an equation that extends this conceptual clarity to include population size change and thereby provides a formal and exact definition for joint ecological and evolutionary change.

BOX 1 The Price equation is an abstract formula to represent evolutionary change. Formulated originally in the early 1970s by George Price (Price 1970, 1972), it postulates some basic properties that all evolutionary systems must satisfy: change over time, ancestor and descendant relations, and a character or phenotype (Rice 2004). Using simple algebraic language, the Price equation represents evolutionary change with the predominant notation,

$$\bar{w}\Delta \bar{z}=\mathrm{Cov}\left(w,z\right)+E\left(w\Delta z\right)$$

In the above equation, $\Delta \bar{z}$ is the change in the average character value z over a time step of arbitrary length, w is an individual's fitness, and $\bar{w}$ average population fitness. On the right‐hand side of the equation, the first term is the covariance between a character value z and fitness w, which reflects $\bar{z}$ change attributable to differential survival and reproduction. The second term is the expected value of wΔz, which reflects the extent to which offspring deviate from parents in z (Rice 2004; Okasha 2006; Frank 2012). A more specific version of the covariance term was already known within the quantitative and population genetics tradition (Robertson 1966), usually representing the action of natural selection. The Price equation adds an expectation term and abstracts away from any specific mechanisms of replication or reproduction, or mechanisms of inheritance. Its definitional nature and lack of substantive biological assumptions has been portrayed both as a strength (Rice 2004; Frank 2012; Luque 2017; Baravalle and Luque 2022), and its greatest weakness. The abstract nature of the Price equation places it at the top of the hierarchy of fundamental theorems of evolution from which the rest (Robertson's theorem, Fisher's fundamental theorem, breeder's equation, Hamilton's rule, adaptive dynamics, etc.) can be derived (Lehtonen 2016, 2018, 2020; Queller 2017). This abstractness is also key to developing a more general view of evolution (Rice 2020; Luque and Baravalle 2021; Edelaar et al. 2023). In contrast, some researchers consider the Price equation just a triviality (even tautological), and useless without further modelling assumptions (van Veelen 2005; van Veelen et al. 2012). The debate remains open (van Veelen 2020; Baravalle et al. 2025).

In biological populations, ecological change is caused by the same processes of individual birth and death that cause evolutionary changes in allele frequencies and phenotypes (Turchin 2001; Connor and Hartl 2004; Barfield et al. 2011). As with evolution, a fundamental equation can exhaustively and exactly define population change. Unlike the Price equation, this fundamental equation is perhaps self‐evident. Population change is simply the addition of individuals minus the removal of individuals from current population size ($N_{t}$), which recovers the new population size ($N_{t+1}$; Box 2). By definition, the relationship $N_{t+1}=N_{t}+\text{Births}-\text{Deaths}$ applies to any closed population. Turchin (2001) argues that general principles are needed to establish a logical foundation for population ecology, and this simple birth and death model and the consequences that logically follow from it (e.g., exponential population growth) are fundamental to population ecology. Any unifying definition of joint ecological and evolutionary change must be able, when formalised, to derive both the Price equation and this birth and death model.

BOX 2 The number of individuals in any closed population (N) at any given time (t+1) is determined by the number of individuals at t ($N_{t}$), plus the number of births (Births) minus the number of deaths (Deaths),

$$N_{t+1}=N_{t}+\text{Births}-\text{Deaths}$$

This equation is necessarily true for any closed population. Despite its simplicity, it is a general equation for defining population change and a starting point for understanding population ecology. Turchin (2001) notes that a consequence of this fundamental equation is the tendency for populations to grow exponentially (technically geometrically in the above case where time is discrete). This inherent underlying tendency towards exponential growth persists even as the complexities of real populations, such as structure, stochasticity, or density‐dependent effects are added to population models (Turchin 2001). Given the assumption that all individuals in the population are identical, a per capita rate of birth, $\text{Birth}s_{t}=b_{t}N_{t}$, and death, $\text{Death}s_{t}=d_{t}N_{t}$, can be defined. Rearranging and defining $\lambda_{t}=1+b_{t}-d_{t}$ gives, $N_{t+1}=N_{t}\lambda_{t}$. Here $\lambda_{t}$ is the finite rate of increase (Gotelli 2001), and note that because $0\leq d_{t}\leq 1$, $\lambda_{t}\geq 0$. Verbally, the change in size of any closed population equals its existing size times its finite rate of increase.

The union of ecological and evolutionary processes has long been recognised (e.g., Darwin 1859; Fisher 1958; Pelletier et al. 2009), but the rise of eco‐evolutionary models, which incorporate both, is relatively recent following a widespread recognition that ecology and evolution can happen on similar timescales (Govaert et al. 2019; Yamamichi et al. 2023). Currently, a universally recognised formal definition of eco‐evolutionary change is lacking, with some theoreticians broadly interpreting “eco‐evolutionary dynamics” to allow for a separation of ecological and evolutionary timescales (Lion et al. 2023) and others advocating for a more narrow interpretation in which no such separation is permitted (Bassar et al. 2021). Bassar et al. (2021) identify two types of eco‐evolutionary models that follow from these interpretations. The first type uses separate equations to model population size change versus evolutionary change, thereby allowing for any number of ecological, evolutionary, or environmental feedbacks (e.g., Lion 2018; Patel et al. 2018; Lion et al. 2023). The second type models population demographics as functions of quantitative traits with ecological and evolutionary change following from demographic processes and trait distributions operating on the same timescale (e.g., Barfield et al. 2011; Simmonds et al. 2020; Jaggi et al. 2024). Both model types can be very general, but like all predictive models, they rely on simplifying assumptions for tractability (Levins 1966; Luque 2017). These simplifying assumptions are often grounded in the Price equation to demonstrate accuracy and logical consistency when modelling evolutionary change (e.g., Coulson and Tuljapurkar 2008; Barfield et al. 2011; Rees and Ellner 2016; Lion 2018). For example, Barfield et al. (2011) link their model back to Price (1970), which they consider to be a “universal law of evolution”, to place their conclusions concerning stage‐structured evolution in the broader context of evolutionary theory. The role of fundamental equations is therefore important for unifying theory (Luque and Baravalle 2021), and we believe that a fundamental equation of eco‐evolutionary change has been curiously overlooked.

We present an equation from which the fundamental equations of ecology and evolutionary biology can be derived. Derivation follows by adding assumptions that are specific to population ecology or evolution in the same way that key equations of population genetics or quantitative genetics can be derived from the Price equation by restricting the domain of interest (e.g., to allele frequencies in the case of population genetics, or to continuous phenotypes in the case of quantitative genetics, Queller 2017). We propose our equation as a formal definition of eco‐evolutionary change.

## 2 A Foundation for Biological Evolution and Population Ecology

To unify biological evolution and population ecology, we must reconcile the Price equation (Box 1) with the general equation for population change (Box 2). The Price equation is critical for partitioning different components of biological change (Price 1970; Frank 1997; Gardner 2008; Luque 2017; Queller 2017; Lehtonen 2018, 2020). It has also been highly useful for integrating evolutionary theory across disciplines (Fox 2006; Brantingham 2007; MacCallum et al. 2012; Frank 2015; Borgstede and Luque 2021; Godsoe et al. 2021; Ulrich et al. 2024). These properties would seem to make it an intuitive starting point for a logical foundation of ecology and evolution, perhaps through some kind of mathematical equivalence (Page and Nowak 2002) or addition of terms (Collins and Gardner 2009), or through the use of its recursive structure (Kerr and Godfrey‐Smith 2009; Frank 2012). But despite its flexibility, the Price equation still relies on relative frequencies, which must by definition sum to one (Frank 2015). This is because the Price equation describes the average change in a population; the frequency of entities is scaled thereby conserving total probability (Frank 2015, 2016). But to recover the fundamental principle of exponential population growth (Turchin 2001), this scaling must be avoided.

We therefore begin with the most fundamental axioms underlying the ecology and evolution of living systems (Rice 2004; Rice and Papadopoulos 2009). In such systems, diversity is discontinuous, in the sense that living systems are composed of discrete entities including individual organisms and groups of organisms (Dobzhansky 1970). Our framework is general enough that entities can be anything discrete, but we will focus on each entity i as an individual organism. Change occurs between the current time step t and a future time step t+1, and time steps can be arbitrarily short or long. Let $\beta_{i}$ be the count of direct descendants of i at t+1 (e.g., offspring if a time step is a single generation). Similarly, let $\delta_{i}$ be the count of deaths summed across i and any of its descendants from t to t+1. For example, if i and all of its descendants persist at t+1, then $\delta_{i}=0$, or if a time step is one season in an annual species, then $\delta_{i}=1$. All individuals are defined by some characteristic $z_{i}$, and $\Delta z_{i}$ defines any change in $z_{i}$ from t to t+1. Together, $z_{i}+\Delta z_{i}$ is the average characteristic across any individual and its descendants alive at t+1. The total number of individuals in the population is N. From this foundation, we define Ω (which takes the same measurement units as z) to be the sum of characteristic values in t+1,

(1) $$\Omega =\sum_{i=1}^{N}\left(\beta_{i}-\delta_{i}+1\right)\left(z_{i}+\Delta z_{i}\right)$$

The foundation of eco‐evolutionary change defined by Equation (1) is therefore a statistical interaction between the demographic processes of birth and death ($\beta_{i}-\delta_{i}+1$) and individual characteristics ($z_{i}+\Delta z_{i}$). From Equation (1), we can derive the most fundamental equations of population ecology and evolutionary biology through an appropriate interpretation of z. Under more limited interpretations of z, we can also interpret Ω as a metric of ecosystem function.

## 3 Population Ecology

To recover the general equation for population ecology (Box 2), we define $z_{i}$ as the identity of i belonging to the population. In other words, we set $z_{i}=1$ to indicate that i is one member of the population and therefore contributes one unit to the total population size. In this restricted case, z is a count, which takes the unit 1 (note that ‘individual’ is a label, not a unit, see Newell and Tiesinga 2019). Further, we assume that individual membership and the unit contribution to population size does not change by setting $\Delta z_{i}=0$. In this case,

$$\Omega =\sum_{i=1}^{N}\left(\beta_{i}-\delta_{i}+1\right).$$

We can now interpret Ω as the population size at t+1, $N_{t+1}$. Summing up $\beta_{i}$, $\delta_{i}$, and current individuals tallies up the total number of individuals in the next time step,

(2) $$N_{t+1}=N_{t}+\text{Births}-\text{Deaths}$$

Using the classical assumptions of population ecology (Box 2, Gotelli 2001), we can then recover the fundamental tendency for populations to grow (or decline) exponentially (Turchin 2001).

## 4 Evolutionary Biology

Recovering the Price equation requires a few more steps. We start by defining individual fitness,

(3) $$w_{i}=\beta_{i}-\delta_{i}+1$$

In this definition, the longevity of the individual matters. All else being equal, an individual that survives from t to t+1 has a higher fitness than one that dies, even if both have the same reproductive output. With this definition of fitness (Equation 3), we substitute $w_{i}$ into Equation (1),

(4) $$\Omega =\sum_{i=1}^{N}\left(w_{i}z_{i}+w_{i}\Delta z_{i}\right)$$

We break Equation (4) down further and multiply each side by 1/N,

(5) $$\frac{1}{N}\Omega =\frac{1}{N}\sum_{i=1}^{N}\left(w_{i}z_{i}\right)+\frac{1}{N}\sum_{i=1}^{N}\left(w_{i}\Delta z_{i}\right)$$

We rewrite the terms on the right‐hand side of Equation (5) as expected values and remove the subscripts,

(6) $$\frac{1}{N}\Omega =E\left(\mathrm{wz}\right)+E\left(\mathrm{w\Delta z}\right)$$

Now we must consider the total conservation of probability (Frank 2015, 2016). In Equation (6), Ω is the total sum trait values ($z_{i}$) across the entire population at t+1 divided by the number of individuals (N) in the population at t. But the size of the population can change from t to t+1. To recover mean trait change for the Price equation (and therefore conserve total probability), we need to account for this change in population size. We cannot treat Ω/N as the mean of z at t+1 (${\bar{z}}^{\prime }$) because we need to weight N by the mean fitness of the population at t to account for any change in population size from t to t+1. We need to multiply the mean trait value ${\bar{z}}^{\prime }$ (at t+1) by the mean fitness $\bar{w}$ (at t) to recover the mean contribution of the N individuals at t to the total Ω (Case and Taper 2000; Ewens 2014). Consequently,

(7) $$\Omega =N\bar{w}\;{\bar{z}}^{\prime }$$

Equation (7) conserves the total probability and recovers Ω as the summed trait value, which has the same measurement units as z and equals expected population growth at t times mean trait value at t+1. This is consistent with the population ecology derivation from the previous section where $z_{i}=1$ by definition, and $\Omega =N_{t+1}$. We can therefore rewrite Equation (6),

(8) $$\bar{w}\;{\bar{z}}^{\prime }=E\left(\mathrm{wz}\right)+E\left(w\Delta z\right)$$

We can rearrange Equation (8) to derive the Price equation by expressing covariance as $\mathrm{Cov}\left(X,Y\right)=E\left(\mathrm{XY}\right)-E\left(X\right)E\left(Y\right)$, and therefore $E\left(\mathrm{XY}\right)=\mathrm{Cov}\left(X,Y\right)+E\left(X\right)E\left(Y\right)$. Substituting into Equation (8),

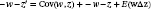




From here,

$$\bar{w}\left({\bar{z}}^{\prime }-\bar{z}\right)=\mathrm{Cov}\left(w,z\right)+E\left(w\Delta z\right)$$

Since $\Delta \bar{z}=\left({\bar{z}}^{\prime }-\bar{z}\right)$

(9) $$\bar{w}\Delta \bar{z}=\mathrm{Cov}\left(w,z\right)+E\left(w\Delta z\right)$$

Equation (9) is the Price equation. From Equation (1), which describes fundamental birth and death processes in a population, we can therefore derive both the most fundamental model of population ecology (Equation 2; Box 2) and the fundamental equation of evolution (Equation 9; Box 1).

## 5 Ecosystem Function

In some cases, Ω could also be interpreted as the total contribution of a population to ecosystem function. This is restricted to cases in which z is a characteristic defining an absolute quantity measured at the whole organism level such as biomass, seed production, carbon capture, flower visits, or nutrient consumption (Collins and Gardner 2009). In such cases, the sum across individuals gives a meaningful total quantity for the population (e.g., the total biomass or seeds produced in the population). When z is instead defined by relative organism‐level measurements such wing loading, nutrient ratio, or diet composition, or when z is measured at a level of biological organisation below the organism (e.g., average cell volume or leaf surface area), Ω does not have a clear population‐level interpretation. In such cases, Equation (1) still defines eco‐evolutionary change; the interpretation of Ω by itself is just not as interesting, biologically. Box 3 provides an instructive example of three plants with different fitnesses and fruit masses.

BOX 3 As an instructive example of our framework, consider a population of $N_{t}=3$ annual plants in which individual total fruit mass (kg) is measured, and change is observed over a year. For all plants, $\delta_{i}=1$ (because annual plants die between t and t+1), and let plant fecundities be $\beta_{1}=1$, $\beta_{2}=1$, and $\beta_{3}=2$. Applying Equation (1) to population change such that $\Omega =N_{t+1}$, $z_{i}=1$ and $\Delta z_{i}=0$, $N_{t+1}=\left(1-1+1\right)\left(1+0\right)+\left(1-1+1\right)\left(1+0\right)+\left(2-1+1\right)\left(1+0\right)=4$ (note $\bar{w}=4⁄3$, so $N_{t}\bar{w}=3\left(4⁄3\right)=4$). Focusing next on the characteristic of total fruit mass, let $z_{1}=0.8$ kg, $z_{2}=1.0$ kg, and $z_{3}=1.5$ kg. Also let $\Delta z_{i}=0.1$ for all plants to reflect a change in soil environment from t to t+1. In this case, population fruit yield at t+1 is $\Omega =\left(1-1+1\right)\left(0.8+0.1\right)+\left(1-1+1\right)\left(1.0+0.1\right)+\left(2-1+1\right)\left(1.5+0.1\right)=5.2$ kg. At t, mean fruit yield per plant was $\left(0.8+1.0+1.5\right)/3=1.1$ kg, but at t+1, mean fruit yield per plant is $\left[\left(0.8+0.1\right)+\left(1.0+0.1\right)+2\left(1.5+0.1\right)\right]⁄4=1.3$ kg. Note that 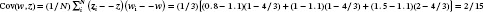 and $E\left(\mathrm{w\Delta z}\right)=4/3\times 0.1=2/15$, so applying the Price equation, $\bar{w}\Delta \bar{z}=2⁄15+2⁄15=4⁄15$. Since $\bar{w}=4⁄3$, multiplying both sides of the equation by 3/4 returns $\Delta \bar{z}=0.2$, which is the mean difference in fruit yield between t+1 and t. The framework expressed in Equation (1) thereby links population change, evolutionary change, and ecosystem function.

## 6 Discussion

An important aspect of scientific progress is the ability to connect disparate theories and models to show how specific empirical and theoretical models are logical (mathematical) consequences of more fundamental ones (Nagel 1961; Morrison 2000). Rather than making simplifying assumptions, as is the approach for specific ecological and evolutionary models, we focus on fundamental axioms that are universal to closed biological systems: discrete individuals, birth, death, and change over time. We define an abstract sum (Ω), to which all individuals in the population contribute. From the basic axiom that each individual is one member of a specific population ($z_{i}=1$ and $\Delta z_{i}=0$), we recover the most general equation of population ecology (Box 2). By defining individual fitness ($w_{i}$) and applying the total conservation of probability to individual frequencies (Frank 2015, 2016), we recover the most fundamental equation of evolution, the Price equation (Box 1). Our Equation (1) thereby provides a foundation for defining eco‐evolutionary change in any population.

The Price equation provides a complete and exact description of evolution in any closed evolving system (Price 1970; Frank 2012). It is derived by rearranging the mathematical notation defining changes in the frequencies and characteristics of any type of entity (e.g., individuals, alleles, Price 1970; Gardner 2008; Luque 2017). This derivation partitions total characteristic change into different components, making it possible to isolate evolutionary mechanisms (e.g., selection) and levels of biological organisation (e.g., group, individual, Frank 1995, 2012; Kerr and Godfrey‐Smith 2009; Luque 2017; Okasha and Otsuka 2020). Because of its abstract nature and lack of any system‐specific assumptions, the Price equation is not dynamically sufficient and makes no predictions about what will happen in any particular system (Gardner 2020). Its role is not to predict, but to formally and completely define and separate components of evolutionary change (Baravalle et al. 2025). The same is true of the general equation for population change (Equation 2), at least as we have used it here where it serves to define what population change means in ecology. This equation formally and completely describes population change in terms of births and deaths. In Equation (1), we therefore have a fundamental equation from which we can derive complete ecological and evolutionary change in any closed biological population. Like all fundamental equations, our equation is necessarily abstract and not dynamically sufficient. We believe that it will be useful for eco‐evolutionary theory in a similar way that the Price equation is useful for evolutionary theory: potentially facilitating specific model development and identifying new conceptual insights, unresolved errors, and sources of model disagreements (see below and Appendix S1).

Our unification recovers the equivalence between the finite rate of increase λ (Box 2) and population mean evolutionary fitness $\bar{w}$ (Box 1). The population growth equation $N_{t+1}=N_{t}\lambda$ can always be rewritten as $N_{t+1}=N_{t}\bar{w}$. This specific equivalence has been proposed before (e.g., Lande 1976), as has the broader relationship between population growth rate and evolutionary fitness (e.g., Fisher 1930; Charlesworth 1980; Lande 1982; Case and Taper 2000; Roff 2008; Lion 2018). We show this from first principles and clarify the relationship between fitness and population growth. Over an arbitrary length of time, fitness is properly defined as $w_{i}=\beta_{i}-\delta_{i}+1$. Rates of change in ecology and evolution are reflected in the first and second statistical moments of fitness, respectively. Population growth rate reflects mean fitness $\bar{w}$, while the rate of evolutionary change reflects the variance in fitness $\mathrm{Var}\left(w\right)⁄\bar{w}$ (i.e., Fisher's fundamental theorem, Frank 1997; Rice 2004; Queller 2017).

Our unification may also help explain, at least partially, some of the success of classical population genetic models. For decades, population genetics (and to some extent quantitative genetics) has been accused of being a reductionist view of evolution, reducing everything to changes in allele frequencies and abstracting away from individuals and their environments (the ecological interactions, MacColl 2011). This has been a line of argumentation by some defenders of the so‐called Extended Synthesis (Pigliucci 2009), especially in relation to niche construction (Odling‐Smee et al. 2003). Famously, Mayr (1959) characterised population genetics as a simple input and output of genes, analogous to “the adding of certain beans to a beanbag and the withdrawing of others” (also called “beanbag genetics”). Historical critics of population genetics could not articulate a clear explanation for why it works so well despite all of its idealisations and simplifications. From the Price equation, we are able to recover classical population and quantitative genetic models (Queller 2017) and develop new ones (Rice 2004, 2020; Luque 2017; Lion 2018). Our Equation (1) contains ecology at its core, and we show how the Price equation logically follows from it after accounting for absolute population growth (Equation 7). We therefore conclude that population and quantitative genetic equations contain ecology (no matter how hidden), and the ecological nature of evolution is implicit in population and quantitative genetic models.

We have focused on the dynamics of a closed population, and in doing so leave ecological and evolutionary change attributable to migration for future work. In population ecology, immigration and emigration can be incorporated by adding a term for each to the right‐hand side of the equation in Box 2 (Gotelli 2001). In evolution, because the Price equation relies on mapping ancestor–descendant relationships, accounting for migration is more challenging. Kerr and Godfrey‐Smith (2009) demonstrate how the Price equation can be extended to allow for arbitrary links between ancestors and descendants, thereby extending the Price equation to allow for immigration and emigration. Frank (2012) presents a simplified version of Kerr and Godfrey‐Smith (2009) that allows some fraction of descendants to be unconnected to ancestors. In both ecology and evolution, accounting for migration is done through the use of additional terms on the right‐hand side of the equations.

We believe our fundamental equation to be complete and exact for any closed population. It therefore implicitly includes any effects of density dependence on population growth (see Box 2), or any social effects on evolutionary change (see Box 1). Both of these effects can be made explicit by specifying how other individuals in a population affect the birth and death of a focal individual. We demonstrate this by deriving more specific models of density‐dependent population growth and multi‐level selection in Appendix S1.

We have shown that we can derive the fundamental equations of population ecology and biological evolution from a single unifying equation. Lastly, we propose our Equation (1) as a potential starting point for defining ecosystem function and further conceptual unification between ecology, evolution, and ecosystem function. The Price equation has previously been used to investigate ecosystem function (Loreau and Hector 2001; Fox 2006), but not with any attempt towards conceptual unification with evolutionary biology. For example, Fox (2006) applied the abstract properties of the Price equation to partition total change in ecosystem function into separate components attributable to species richness, species composition, and context dependent effects. This approach provides a framework for comparing the effects of biodiversity on ecosystem function in empirical systems (Fox 2006; Winfree et al. 2015; Mateo‐Tomás et al. 2017). Instead, our Equation (1) defines Ω as total ecosystem function contributed by a focal population. It is therefore possible to investigate ecological, evolutionary, and ecosystem function change from the same shared framework.

Over 120 years ago, Needham (1904) described natural history as “the study of the phenomena of fitness”. Fundamentally, we show why eco‐evolutionary change is a statistical interaction between fitness and individual characteristics. From this definition, we can recover both population size change and evolutionary change.

## Author Contributions

**A. Bradley Duthie:** conceptualization (equal), data curation (equal), formal analysis (equal), funding acquisition (lead), investigation (equal), methodology (equal), resources (equal), software (equal), supervision (equal), validation (equal), visualization (equal), writing – original draft (lead), writing – review and editing (equal). **Victor J. Luque:** conceptualization (equal), data curation (equal), formal analysis (equal), funding acquisition (supporting), investigation (equal), methodology (equal), project administration (equal), resources (equal), software (equal), supervision (equal), validation (equal), visualization (equal), writing – original draft (supporting), writing – review and editing (equal).

## Conflicts of Interest

The authors declare no conflicts of interest.

## Supporting information


**Appendix S1:** Supporting Information.

## Data Availability

The authors have nothing to report.
