# Supplementary material for: Foundations of Ecological and Evolutionary Change
Source: Ecol Evol. 2025 Nov 12;15(11):e72454. doi: 10.1002/ece3.72454 (PMC12611352; doi:10.1002/ece3.72454)
Supplement: Supplementary file 1 — Appendix S1: Supporting Information. [file ECE3-15-e72454-s001.pdf]

Foundations of ecological and evolutionary change: Supporting Information 1

A. Bradley Duthie[1,a,*] and Victor J. Luque[2,a]

[1] Department of Biological and Environmental Sciences, University of Stirling, Stirling, Scotland [2] Department of Philosophy, University of Valencia, Valencia, Spain [*] Corresponding author: alexander.duthie@stir.ac.uk [a] Equal contribution

This supporting information demonstrates how to derive well-established models and develop new theory in population ecology and evolutionary biology from equation 1 in the main text,

$$\Omega = \sum_{i=1}^{N} \left(\beta_i - \delta_i + 1\right)\left(z_i + \Delta z_i\right). \tag{1}$$

In the main text, we derived both the Price equation and the birth-death model from the above. Here we (1) integrate interactions between individuals to recover density-dependent population growth, (2) integrate groups within the population to recover multi-level selection, and (3) integrate both to model a system in which multi-level selection and density-dependent population change occur simultaneously. Finally, we (4) expand on our derivation of multi-level selection to demonstrate how it might be used to recover both community and population-level evolutionary change, partitioning this change into different components.

1. Density-dependent population growth

There are two potential ways to model the incorporation of density dependence into population growth. We start with what is likely to be the most familiar model to readers, which focuses on individual growth rate r_i. We then use a slightly different model focusing on fitness w_i. First, note that here we set $\Omega = N_{t+1}$, and $z_i = 1$ and $\Delta z_i = 0$ for all individuals as in the main text. We can define $r_i = \beta_i - \delta_i$ as the individual growth rate for i (Lion 2018; Lion, Sasaki, and Boots 2023). In this case,

$$N_{t+1} = \sum_{i=1}^{N_t} \left(r_i + 1\right) \tag{S1.1}$$

Mathematically, the most general approach here would be to define individual growth as a function of the entire system $\mathbf{E}$, $r_i(\mathbf{E})$, where $\mathbf{E}$ is a vector with elements including any parameters potentially relevant to r_i. Taking this approach would recover a version of eqn S1.1 in Lion (2018) and permit any relationship between the system and a focal individual's growth. Limiting our focus to the effects of other individuals (j) and assuming that the effects of these individuals are additive, let $a_{ij|\cdot}$ be the effect of individual j on the growth rate attributable to i conditioned on all other individuals within the population such that $r_i\left(1 - \sum_{i=j}^{N} a_{ij|\cdot}\right)$ defines the realised growth rate of i,

$$N_{t+1} = \sum_{i=1}^{N_t} \left(r_i \left(1 - \sum_{i=j}^{N_t} a_{ij|\cdot}\right) + 1\right). \tag{S1.2}$$

Assuming that individual effects of j on i are also independent, we can remove the condition in S1.2,

$$N_{t+1} = \sum_{i=1}^{N_t} \left(r_i \left(1 - \sum_{i=j}^{N_t} a_{ij} \right) + 1 \right).$$

Further assuming that all individuals have the same per capita effect such that $a = a_{ij}$ for any i and j pair (as might be reasonable given resource competition in a well-mixed population),

$$N_{t+1} = \sum_{i=1}^{N_t} \left(r_i \left(1 - aN_t \right) + 1 \right).$$

If r_i values are identical,

$$N_{t+1} = N_t + rN_t \left(1 - aN_t \right). \quad \text{(S1.3)}$$

Equation S1.3 therefore recovers a classic version of a discrete time logistic growth by making assumptions from an exact model of eco-evolutionary change.

An alternative approach would be to model the effects of an individual j on the fitness of i (w_i), thereby replacing eqn S1.1 with $N_{t+1} = \sum_{i=1}^{N_t} w_i$ and replacing eqn S1.2 with,

$$N_{t+1} = \sum_{i=1}^{N_t} w_i \left(1 - \sum_{i=j}^{N_t} \alpha_{ij|\cdot} \right).$$

Note that we have used α_{ij} to represent the effect of j on the fitness of i for clarity in the sections below. By making the same assumptions of additivity, independence, and identical effects such that $\alpha = \alpha_{ij}$ for all j on i, and assuming fitness is equal ($w_i = \bar{w}$), we can derive,

$$N_{t+1} = \bar{w} N_t (1 - \alpha N_t). \quad \text{(S1.4)}$$

Hence, S1.4 is an alternative way to express logistic growth.

2. Multi-level selection

We can recover multi-level selection from our eqn 1. Here we derive the original form of the multi-level Price (1972) equation as it appears in eqn 3.1 of Lehtonen (2020). Individuals belong to one of K total groups where j indexes K groups and i indexes individuals. Individuals do not overlap in group membership. The size of group j is denoted as N_j. Equation S2.1 below uses summations to partition how individuals within each group contribute to Ω,

$$\Omega = \sum_{j=1}^{K} \sum_{i=1}^{N_j} \left(\beta_{ji} - \delta_{ji} + 1 \right) \left(z_{ji} + \Delta z_{ji} \right). \quad \text{(S2.1)}$$

In S2.1, indices β_{ji}, δ_{ji}, and z_{ji} identify individual i in group j. We set $w_{ji} = \beta_{ji} - \delta_{ji} + 1$, and for simplicity let $\Delta z_{ji} = 0$ (i.e., no transmission bias, but see section 4 below),

$$\Omega = \sum_{j=1}^{K} \sum_{i=1}^{N_j} w_{ji} z_{ji}.$$

For ease of presentation, with no loss of generality, we assume all group sizes are equal with a group size of $N_j = n$ for all j. If group sizes differ, then weighted expectations and covariances are instead needed (Lehtonen 2020). Given equal group sizes, the total number of individuals (N) equals $K \times n$, and,

$$\frac{\Omega}{Kn} = \left(\frac{1}{K}\right)\left(\frac{1}{n}\right)\sum_{j=1}^{K}\sum_{i=1}^{n} w_{ji}z_{ji}.$$

Rearranging,

$$\frac{\Omega}{Kn} = \frac{1}{K}\sum_{j=1}^{K}\frac{1}{n}\sum_{i=1}^{n} w_{ji}z_{ji}.$$

The inner summation can be rewritten as an expectation for group j,

$$\frac{\Omega}{Kn} = \frac{1}{K}\sum_{j=1}^{K}\mathrm{E}_j\left(w_{ji}z_{ji}\right).$$

As in the main text, we note $\mathrm{E}(XY) = \mathrm{Cov}(X,Y) + \mathrm{E}(X)\mathrm{E}(Y)$. Defining $Cov_j(w_j, z_j)$ as the covariance between w_{ji} and z_{ji} for group j,

$$\frac{\Omega}{Kn} = \frac{1}{K}\sum_{j=1}^{K}\left(\mathrm{Cov}_j\left(w_{ji}, z_{ji}\right) + \mathrm{E}_j\left(w_{ji}\right)\mathrm{E}_j\left(z_{ji}\right)\right).$$

We can separate the summation for each term,

$$\frac{\Omega}{Kn} = \frac{1}{K}\sum_{j=1}^{K}\left(\mathrm{Cov}_j\left(w_{ji}, z_{ji}\right)\right) + \frac{1}{K}\sum_{j=1}^{K}\left(\mathrm{E}_j\left(w_{ji}\right)\mathrm{E}_j\left(z_{ji}\right)\right).$$

Using the notation $\bar{w}_j = E_j(w_{ji})$ and $\bar{z}_j = E_j(z_{ji})$ to indicate the expectation in group j,

$$\frac{\Omega}{Kn} = \left(\frac{1}{K}\sum_{j=1}^{K}\mathrm{Cov}_j\left(w_{ji}, z_{ji}\right)\right) + \mathrm{E}\left(\bar{w}_j\bar{z}_j\right).$$

We can also rewrite the first term on the right-hand side as an expectation,

$$\frac{\Omega}{Kn} = \mathrm{E}\left(\mathrm{Cov}_j\left(w_j, z_j\right)\right) + \mathrm{E}\left(\bar{w}_j\bar{z}_j\right).$$

We can rearrange the second term on the right-hand side ($\bar{\bar{w}}$ indicates grand mean over all groups),

$$\frac{\Omega}{Kn} = \mathrm{E}\left(\mathrm{Cov}_j\left(w_j, z_j\right)\right) + \mathrm{Cov}\left(\bar{w}_j, \bar{z}_j\right) + \bar{\bar{w}}\bar{\bar{z}}.$$

As in the main text, note that $Kn\bar{\bar{w}}$ accounts for differences in total population size from t to $t+1$, with $\bar{\bar{w}}$ being mean fitness across all groups. We can therefore set $\Omega = Kn\bar{\bar{w}}\bar{\bar{z}}'$, so,

$$\frac{Kn\bar{\bar{w}}\bar{\bar{z}}'}{Kn} - \bar{\bar{w}}\bar{\bar{z}} = \mathrm{E}\left(\mathrm{Cov}_j\left(w_j, z_j\right)\right) + \mathrm{Cov}\left(\bar{w}_j, \bar{z}_j\right).$$

Because $\Delta\bar{\bar{z}} = \bar{\bar{z}}' - \bar{\bar{z}}$,

$$\bar{\bar{w}}\Delta\bar{\bar{z}} = \mathrm{Cov}\left(\bar{w}_j, \bar{z}_j\right) + \mathrm{E}\left(\mathrm{Cov}_j\left(w_j, z_j\right)\right). \tag{S2.2}$$

Equation S2.2 therefore recovers the multi-level Price (1972) equation (Lehtonen 2020) from a starting point of eco-evolutionary change in different groups. Equation S2.2 can be found in Lehtonen (2016) B2.I, who then derives a multi-level selection version of Hamilton's rule predicting the evolution of altruism.

3. Integration of ecology and evolution

For simplicity, we now focus on showing an integration between ecology and evolution using a population with no multi-level selection, and we let $\Delta z_i = 0$ (i.e., no transmission bias). As above in the section on density-dependent population growth, we define $w_i = \beta_i - \delta_i + 1$ and use α_{ij} to represent the effect of individual j on the fitness of individual i. Our starting equation is therefore,

$$\Omega = \sum_{i=1}^{N} w_i \left(1 - \sum_{j=1}^{N} \alpha_{ij}\right) z_i. \tag{S3.1}$$

We have already demonstrated that if we assume all individuals have the same effect on a focal individual such that $\alpha = \alpha_{ij}$ for all i and j pairs, we can recover equation S1.4 when $z_i = 1$ and Ω is therefore interpreted as the count of entities,

$$N_{t+1} = \bar{w}N_t\left(1 - \alpha N_t\right).$$

We now start from S3.1 to derive $\Delta\bar{z}$. The objective is to use our definition of eco-evolutionary change to simultaneously recover how interactions between individuals affect population change and evolutionary change.

We start by dividing both sides of S3.1 by N,

$$\frac{\Omega}{N} = \frac{1}{N}\sum_{i=1}^{N} w_i \left(1 - \sum_{j=1}^{N} \alpha_{ij}\right) z_i. \tag{S3.2}$$

We can express the right-hand side of eqn S3.2 as an expectation,

$$\frac{\Omega}{N} = \mathrm{E}\left(w_i \left(1 - \sum_{j=1}^{N} \alpha_{ij}\right) z_i\right).$$

We can rewrite the right-hand side in terms of covariances,

$$\frac{\Omega}{N} = \mathrm{Cov}\left(w_i\left(1 - \sum_{j=1}^{N}\alpha_{ij}\right), z_i\right) + \mathrm{E}\left(w_i\left(1 - \sum_{j=1}^{N}\alpha_{ij}\right)\right)\mathrm{E}\left(z_i\right). \tag{S3.3}$$

The expectations in the second term on the right-hand side of eqn S3.3 can be replaced with overbars to represent the mean,

$$\frac{\Omega}{N} = \text{Cov}\left(w_i\left(1-\sum_{j=1}^{N}\alpha_{ij}\right), z_i\right) + \overline{w_i\left(1-\sum_{j=1}^{N}\alpha_{ij}\right)}\bar{z}_i. \quad \text{(S3.4)}$$

In the main text, we noted that $\Omega = N\bar{w}\bar{z}'$. Here mean fitness incorporates individual interactions, therefore,

$$\Omega = N\overline{w_i\left(1-\sum_{j=1}^{N}\alpha_{ij}\right)}\bar{z}'.$$

We can therefore rewrite S3.4,

$$\overline{w_i\left(1-\sum_{j=1}^{N}\alpha_{ij}\right)}\bar{z}' - \overline{w_i\left(1-\sum_{j=1}^{N}\alpha_{ij}\right)}\bar{z}_i = \text{Cov}\left(w_i\left(1-\sum_{j=1}^{N}\alpha_{ij}\right), z_i\right).$$

Noting again $\Delta\bar{z} = \bar{z}' - \bar{z}$,

$$\overline{w_i\left(1-\sum_{j=1}^{N}\alpha_{ij}\right)}\Delta\bar{z} = \text{Cov}\left(w_i\left(1-\sum_{j=1}^{N}\alpha_{ij}\right), z_i\right). \quad \text{(S3.5)}$$

We can rewrite the right-hand side of S3.5,

$$\overline{w_i\left(1-\sum_{j=1}^{N}\alpha_{ij}\right)}\Delta\bar{z} = \text{Cov}\left(w_i - w_i\sum_{j=1}^{N}\alpha_{ij}, z_i\right). \quad \text{(S3.6)}$$

The covariance term in S3.6 can be split without any additional assumptions,

$$\overline{w_i\left(1-\sum_{j=1}^{N}\alpha_{ij}\right)}\Delta\bar{z} = \text{Cov}\left(w_i, z_i\right) - \text{Cov}\left(w_i\sum_{j=1}^{N}\alpha_{ij}, z_i\right). \quad \text{(S3.7)}$$

If we are able to further assume that w_i and the summation over α_{ij} are independent, then we could rewrite eqn S3.7,

$$\overline{w_i\left(1-\sum_{j=1}^{N}\alpha_{ij}\right)}\Delta\bar{z} = \text{Cov}\left(w_i, z_i\right) - \text{Cov}\left(\sum_{j=1}^{N}\alpha_{ij}, z_i\right)\bar{w}_i. \quad \text{(S3.8)}$$

Partitioning fitness into different components with the Price equation is commonplace. But this derivation highlights, e.g., the ecological and evolutionary relationship between nonsocial and social components of fitness, and population size. For example, the second term on the right-hand side of S3.8 shows the covariance between the sum of social interactions and a trait. When traits covary with the interaction between sociality and fitness, they will have a stronger effect on trait change.

4. Expanded derivation of multi-level selection

Here we expand on what we did in this supporting information section 2, deriving multi-level evolutionary change without making the assumption that $\Delta z_{ji} = 0$. Our objective here is slightly different from simply recovering multi-level selection. Here we suggest a way to partition mean trait change in a community into components reflecting biological processes occurring at the species and individual level.

To make this example more instructive, let z_{ji} be the mass of individual i in species j, and suppose that the focus concerns the total biomass in the community. Let there be K species in this community and N_j individuals per species j. We can recover the total biomass Ω of the community at time t by summing up the masses of all individuals i for each population of each species j,

$$\Omega_t = \sum_{j}^{K} \sum_{i}^{N_j} z_{ji}. \tag{S4.1}$$

In eqn S4.1, total biomass at time $t+1$ will be determined by the absolute fitness of each individual $w_{ji} = \beta_{ji} - \delta_{ji} + 1$, where β_{ji} defines the number of births from t to $t+1$ attributable to individual i in species j, and δ_{ji} is indicates individual death ($\delta_{ji} = 1$) or survival ($\delta_{ji} = 0$). Changes in individual mass z_{ji} are defined by Δz_{ji}. Consequently, total biomass at $t+1$ equals,

$$\Omega_{t+1} = \sum_{j}^{K} \sum_{i}^{N_j} w_{ji} \left(z_{ji} + \Delta z_{ji} \right). \tag{S4.2}$$

Again, note that if we instead define z_{ji} as the individual unit $z_{ji} = 1$ (i.e., an individual i contributes a count of 1 to population j) and $\Delta z_{ji} = 0$ (this contribution quantity cannot change), then we recover total community size at $t+1$ because we are summing up all living individuals and their descendants. As in section 2 of this supporting information, for simplicity, assume that each species i has the same number of species, $N_j = n$ for all j. Note that following from eqn S4.2,

$$\Omega_{t+1} = \sum_{j}^{K} \sum_{i}^{n} \left(w_{ji} z_{ji} + w_{ji} \Delta z_{ji} \right).$$

We can divide both sides of the equation by Kn,

$$\frac{\Omega_{t+1}}{Kn} = \left(\frac{1}{K} \right) \left(\frac{1}{n} \right) \sum_{j}^{K} \sum_{i}^{n} \left(w_{ji} z_{ji} + w_{ji} \Delta z_{ji} \right). \tag{S4.3}$$

We can rewrite eqn S4.3,

$$\frac{\Omega_{t+1}}{Kn} = \frac{1}{K} \sum_{j}^{K} \left(\frac{1}{n} \sum_{i}^{n} \left(w_{ji} z_{ji} + w_{ji} \Delta z_{ji} \right) \right).$$

We can separate the inner summation,

$$\frac{\Omega_{t+1}}{Kn} = \frac{1}{K} \sum_{j}^{K} \left(\frac{1}{n} \sum_{i}^{n} \left(w_{ji} z_{ji} \right) + \frac{1}{n} \sum_{i}^{n} \left(w_{ji} \Delta z_{ji} \right) \right).$$

These inner summations can be rewritten as expectations. For this, we will drop the individual subscripts i,

$$\frac{\Omega_{t+1}}{Kn} = \frac{1}{K}\sum_{j}^{K}\left(\mathrm{E}\left(w_j z_j\right) + \mathrm{E}\left(w_j \Delta z_j\right)\right). \tag{7}$$

Because $\mathrm{Cov}(X, Y) = \mathrm{E}(XY) - \mathrm{E}(X)\mathrm{E}(Y)$,

$$\frac{\Omega_{t+1}}{Kn} = \frac{1}{K}\sum_{j}^{K}\left(\mathrm{Cov}\left(w_j, z_j\right) + \mathrm{E}\left(w_j\right)\mathrm{E}\left(z_j\right) + \mathrm{Cov}\left(w_j, \Delta z_j\right) + \mathrm{E}\left(w_j\right)\mathrm{E}\left(\Delta z_j\right)\right).$$

We can separate the summation across K,

$$\frac{\Omega_{t+1}}{Kn} = \frac{1}{K}\sum_{j}^{K}\left(\mathrm{Cov}\left(w_j, z_j\right)\right) + \frac{1}{K}\sum_{j}^{K}\left(\mathrm{E}\left(w_j\right)\mathrm{E}\left(z_j\right)\right) + \frac{1}{K}\sum_{j}^{K}\left(\mathrm{Cov}\left(w_j, \Delta z_j\right)\right) + \frac{1}{K}\sum_{j}^{K}\left(\mathrm{E}\left(w_j\right)\mathrm{E}\left(\Delta z_j\right)\right). \tag{S4.4}$$

We can rewrite terms 2 and 4 on the right-hand side of eqn S4.4 as expectations. To make this easier to follow, we will use the notation $\bar{w}_j = \mathrm{E}(w_j)$ and $\bar{z}_j = \mathrm{E}(z_j)$,

$$\frac{\Omega_{t+1}}{Kn} = \frac{1}{K}\sum_{j}^{K}\left(\mathrm{Cov}\left(w_j, z_j\right)\right) + \mathrm{E}\left(\bar{w}_j \bar{z}_j\right) + \frac{1}{K}\sum_{j}^{K}\left(\mathrm{Cov}\left(w_j, \Delta z_j\right)\right) + \mathrm{E}\left(\bar{w}_j \overline{\Delta z_j}\right). \tag{S4.5}$$

Note that the overbar in S4.5 spans $\overline{\Delta z_j}$ in the last term to indicate that this is the expected change in z_j, and *not* the change in expected z_j (which might be different). From here, we can also get expectations over K for the covariance terms,

$$\frac{\Omega_{t+1}}{Kn} = \mathrm{E}\left(\mathrm{Cov}\left(w_j, z_j\right)\right) + \mathrm{E}\left(\bar{w}_j \bar{z}_j\right) + \mathrm{E}\left(\mathrm{Cov}\left(w_j, \Delta z_j\right)\right) + \mathrm{E}\left(\bar{w}_j \overline{\Delta z_j}\right). \tag{S4.6}$$

Using the definition of covariance, we can expand terms 2 and 4 on the right-hand side of eqn S4.6,

$$\frac{\Omega_{t+1}}{Kn} = \mathrm{E}\left(\mathrm{Cov}\left(w_j, z_j\right)\right) + \mathrm{Cov}\left(\bar{w}_j, \bar{z}_j\right) + \mathrm{E}\left(\bar{w}_j\right)\mathrm{E}\left(\bar{z}_j\right) + \mathrm{E}\left(\mathrm{Cov}\left(w_j, \Delta z_j\right)\right) + \mathrm{Cov}\left(\bar{w}_j, \overline{\Delta z_j}\right) + \mathrm{E}\left(\bar{w}_j\right)\mathrm{E}\left(\overline{\Delta z_j}\right).$$

Again, to make this easier to follow, we will use the notation $\bar{\bar{w}} = \mathrm{E}(\bar{w}_j)$ and $\bar{\bar{z}} = \mathrm{E}(\bar{z}_j)$,

$$\frac{\Omega_{t+1}}{Kn} = \mathrm{E}\left(\mathrm{Cov}\left(w_j, z_j\right)\right) + \mathrm{Cov}\left(\bar{w}_j, \bar{z}_j\right) + \bar{\bar{w}}\bar{\bar{z}} + \mathrm{E}\left(\mathrm{Cov}\left(w_j, \Delta z_j\right)\right) + \mathrm{Cov}\left(\bar{w}_j, \overline{\Delta z_j}\right) + \bar{\bar{w}}\overline{\overline{\Delta z}}. \tag{S4.7}$$

It is now a good time to define Ω_{t+1} in terms of the sum amount of mass, i.e., the total biomass in a community at $t+1$. As in section 2, this will be the product of the grand mean fitness ($\bar{\bar{w}}$) and the grand mean trait of all individuals in the community at $t+1$ ($\bar{\bar{z}}'$). Note that $\bar{\bar{w}}\bar{\bar{z}}'$ is the grand mean fitness at t times the grand mean trait of descendants at $t+1$, which we multiply by the total number of individuals in the community Kn to get $\Omega_{t+1} = Kn\bar{\bar{w}}\bar{\bar{z}}'$. We can therefore rewrite eqn S4.7,

$$\bar{\bar{w}}\bar{\bar{z}}' = \mathrm{E}\left(\mathrm{Cov}\left(w_j, z_j\right)\right) + \mathrm{Cov}\left(\bar{w}_j, \bar{z}_j\right) + \bar{\bar{w}}\bar{\bar{z}} + \mathrm{E}\left(\mathrm{Cov}\left(w_j, \Delta z_j\right)\right) + \mathrm{Cov}\left(\bar{w}_j, \overline{\Delta z_j}\right) + \bar{\bar{w}}\overline{\overline{\Delta z}}. \tag{S4.8}$$

Since $\Delta\bar{\bar{z}} = \bar{\bar{z}}' - \bar{\bar{z}}$, i.e., the change in the grand mean of the trait (mass in this case) is the grand mean at $t+1$ minus the grand mean at t, we can subtract $\bar{\bar{w}}\bar{\bar{z}}$ from both sides of eqn S4.8 and rearrange,

$$\bar{\bar{w}}\Delta\bar{\bar{z}} = \mathrm{E}\left(\mathrm{Cov}\left(w_j, z_j\right)\right) + \mathrm{Cov}\left(\bar{w}_j, \bar{z}_j\right) + \mathrm{E}\left(\mathrm{Cov}\left(w_j, \Delta z_j\right)\right) + \mathrm{Cov}\left(\bar{w}_j, \overline{\Delta z_j}\right) + \bar{\bar{w}}\overline{\overline{\Delta z}}. \tag{S4.9}$$

Finally, we rearrange the terms on the right-hand side of S4.9,

$$\bar{\bar{w}}\Delta\bar{\bar{z}} = \mathrm{Cov}\left(\bar{w}_j, \bar{z}_j\right) + \mathrm{E}\left(\mathrm{Cov}\left(w_j, z_j\right)\right) + \mathrm{Cov}\left(\bar{w}_j, \overline{\Delta z_j}\right) + \mathrm{E}\left(\mathrm{Cov}\left(w_j, \Delta z_j\right)\right) + \bar{\bar{w}}\overline{\overline{\Delta z}}. \tag{S4.10}$$

In S4.10, we have a Price-like equation that describes the mean change in trait across all individuals and species. Note that the first two terms on the right-hand side are the same as found in Lehtonen (2016) and section 2 of this supporting information, which therefore recovers multi-level selection.

More investigation is needed, but an interesting approach might be to consider how the different terms in S4.10 could correspond to ecological and evolutionary processes. For example, we might be able to interpret the first term $\mathrm{Cov}\left(\bar{w}_j, \bar{z}_j\right)$ as the effects of selection and drift at the level of species, i.e., potentially differences in mean fitness (growth rates) of species attributable to differences in a specific trait such as body mass. The second term $\mathrm{E}\left(\mathrm{Cov}\left(w_j, z_j\right)\right)$ could be attributed to selection and genetic drift on body mass within species. The third term $\mathrm{Cov}\left(\bar{w}_j, \overline{\Delta z_j}\right)$ might reflect changes in species fitness that are attributable to expected changes in the mean species trait. This could include demographic change in a population. For example, the average individual in a species might increase in mass due to the changing demographics of a population, so the expected change in total biomass of a species $\overline{\Delta z_j}$ might be correlated the population growth rate of the species. The fourth term is the covariance between the change in body mass and fitness, $\mathrm{E}\left(\mathrm{Cov}\left(w_j, \Delta z_j\right)\right)$. Conceptually, do individuals that experience a change in body mass increase (or decrease) their fitness? This could potentially reflect mutation, individual development, or plasticity. Finally, we have the term $\bar{\bar{w}}\overline{\overline{\Delta z}}$, which is the product between the mean fitness across all individuals in the community and the expected change in a trait for any individual in the community.

Note that some biological processes might be represented by a shift between terms. For example, speciation will likely be represented by some quantity shifting from the second term to the first term. This is because variation within species has, by definition, been lost (a subset of individuals in the population now belong to a new species). But we also have more variation among species (because there are more species with potentially different mean traits). Overall, we believe that this framework introduces new avenues for integrating ecological and evolutionary theory from first principles.

References

Lehtonen, Jussi. 2016. "Multilevel selection in kin selection language." *Trends in Ecology and Evolution* xx: 1–11. https://doi.org/10.1016/j.tree.2016.07.006.

———. 2020. "The Price Equation and the Unity of Social Evolution Theory." *Philosophical Transactions of the Royal Society B: Biological Sciences* 375: 20190362. https://doi.org/10.1098/rstb.2019.0362.

Lion, Sébastien. 2018. "Theoretical approaches in evolutionary ecology: environmental feedback as a unifying perspective." *American Naturalist* 191 (1). https://doi.org/10.1086/694865.

Lion, Sébastien, Akira Sasaki, and Mike Boots. 2023. "Extending Eco-Evolutionary Theory with Oligomorphic Dynamics." *Ecology Letters* 26 (September): S22–46. https://doi.org/10.1111/ele.14183.

Price, George R. 1972. "Extension of covariance selection mathematics." *Annals of Human Genetics* 35 (4): 485–90. https://doi.org/10.1111/j.1469-1809.1957.tb01874.x.
